#  Long-term corticosteroid monotherapy successfully cured seronegative hepatitis B virus-associated glomerulonephritis: a case report with an 8-year follow-up

**DOI:** 10.1007/s11255-020-02673-x

**Published:** 2020-10-13

**Authors:** Yan Zhang, Sha Chen, Ding-Wei Yang

**Affiliations:** 1 Division of Nephrology, Department of Internal Medicine, Tianjin Hospital, No. 406 Jiefang South Road, Hexi District, Tianjin, 300211 China; 2 Graduate School, Tianjin Medical University, Tianjin, China

Editor,

Seronegative hepatitis B virus (HBV)-associated glomerulonephritis (snHBV-GN) is defined as glomerulonephritis with the presence of HBV antigens in the renal tissue in the absence of HBV antigens and detectable HBV-DNA in the serum while excluding other secondary glomerulonephritis [1]. Currently, there are no therapeutic guidelines for snHBV-GN. We report a case of snHBV-GN recovered completely after long-term corticosteroid monotherapy.

**Fig. 1:**
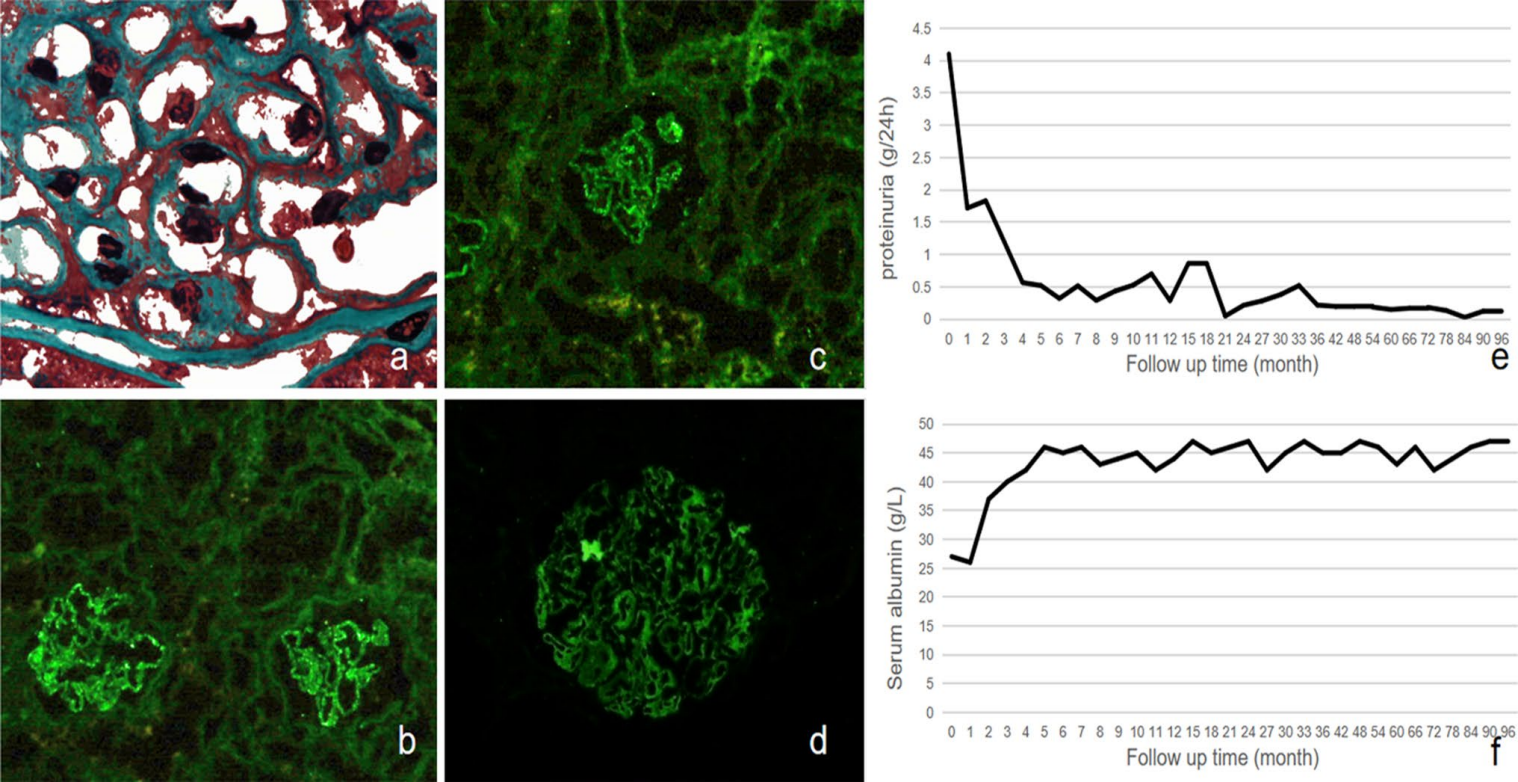
Renal pathological changes and laboratory findings of the patient with sn HBV-GN **a** Light microscope showed GBM thickening (Masson × 600). **b** Immunofluorescent staining showed the deposition of HBsAg in GBM. **c** Immunofluorescent staining showed the deposition of HBcAg in GBM. **d** Immunofluorescent staining showed the deposition of C3 in GBM. **e** Changes in proteinuria levels before and after corticosteroid treatment. **f** The trend of serum albumin before and after corticosteroid treatment. sn HBV-GN, seronegative hepatitis B virus-associated glomerulonephritis. GBM, glomerular basement membrane

A 26-year-old female was admitted for foamy urine for 2 weeks. Physical examination showed: bilateral ankle edema, blood pressure of 125/83 mmHg, and no other abnormalities. Laboratory examination revealed massive proteinuria (4.1 g/24 h), hypoalbuminemia (27 g/L), and mild dyslipidemia, suggesting nephrotic syndrome. Based on renal biopsy results that HBsAg and HBcAg deposited below the capillary epithelium in linear patterns (Fig. 1a–d), alongside negative HBV antigens and undetectable HBV-DNA in the serum (< 500 IU/mL), snHBV-GN was diagnosed. Oral methylprednisolone was given with the starting dose of 0.8 mg/kg/d until serum albumin increased to 30 g/L and then was tapered until 2 mg/d as maintenance dose. The patient’s earliest response was at 2 weeks with proteinuria of 2.125 g/24 h. Complete remission (CR) was achieved at 8 months, with proteinuria of 0.291 g/24 h and serum albumin being 43 g/L. During the 3-year treatment, her symptoms were relieved and proteinuria decreased continuously (4.1 g/24 h vs 0.218 g/24 h) with improved serum albumin (27 g/L vs 45 g/L). Furthermore, she remained in CR for another 5 years after discontinuing corticosteroids, presenting with proteinuria in remission (0.028–0.2 g/24 h) and normal serum albumin (42–47 g/L) (Fig. 1e, f). She conceived four years after discontinuation of treatment. She was in good condition without relapse during pregnancy and had a healthy baby. During the 8-year follow-up, the HBV serological markers remained negative and serum HBV-DNA remained undetectable, while liver and kidney function tests were all within normal limits. Moon face and upper respiratory infection were primary adverse reactions that resolved after cessation of methylprednisolone or adding antibiotics, respectively.

To our best knowledge, this is the first snHBV-GN patient followed up for 8 years who was treated with corticosteroid monotherapy. The patient achieved CR, and maintained in remission for 8 years and gave a birth to a healthy child successfully with no relapse. Relevant studies revealed that for snHBV-GN patients, immunosuppressive agents could also achieve favourable effects and antiviral therapy was nonessential [2–5]. Our results and related studies demonstrated the usage of corticosteroids would not induce serious adverse events in snHBV-GN patients, which indicated corticosteroid monotherapy was safe for snHBV-GN under close monitoring. According to glomerular deposition of HBV antigens along with immunoglobulin and complement components, it can be concluded that immune reaction but not HBV-induced direct injury is the main pathogenesis of snHBV-GN. The presumption was further supported by the favorable outcome obtained by corticosteroid monotherapy.

In conclusion, long-term corticosteroid monotherapy is effective and safe for snHBV-GN. Young female patients with snHBV-GN are able to become pregnant without relapse after remission. Immune reaction might be the main pathogenesis of snHBV-GN.
